# Scalable Solution-Processed Electrolyte Membranes with Optimized Microstructure for High-Performance Protonic Ceramic Electrochemical Cells

**DOI:** 10.1021/acsami.5c16287

**Published:** 2025-12-01

**Authors:** Anshu Kumari, Shuanglin Zheng, Saroj Karki, Idris Temitope Bello, Jiufeng Ruan, Yuqi Geng, Hanping Ding

**Affiliations:** School of Aerospace and Mechanical Engineering, 6187 University of Oklahoma, Norman, Oklahoma 73019, United States

**Keywords:** wet powder spraying, proton-conducting electrochemical cells, thin-film deposition, solution optimization, dense microstructure

## Abstract

Proton-conducting electrochemical cells (PCECs) are promising for efficient hydrogen production, but achieving dense, uniform, thin electrolyte layers remains a key challenge, particularly for scalable fabrication. Here, we present a solution-processed deposition approach with a mechanistically optimized slurry for uniform electrolyte formation. By tailoring particle size distribution, solid loading, and solvent/additive balance, we regulated wetting behavior and evaporation kinetics of the electrolyte slurry to promote homogeneous electrolyte particle packing. These features facilitate tight grain boundary contact and early stage neck growth during sintering, eliminating residual porosity, and improving mechanical integrity. The resulting ∼15 μm thick electrolyte shows high density, strong electrode adhesion, and stable interfaces outperforming the previously reported spray-based fabricated electrolyte by about 31% at 600 °C in FC mode. Single cells deliver 0.962 W cm^–2^ at 600 °C in fuel cell mode and 1.31 A cm^–2^ at 1.3 V in electrolysis mode, maintaining robust performance over 100 h with negligible degradation (≤0.02% h^–1^) in each mode. Scale-up to 2.5 cm diameter substrates confirmed reproducible densification and geometric stability. This work demonstrates a cost-effective, scalable route where control over particle-fluid interactions and drying dynamics enables a superior electrolyte microstructure and high PCEC performance.

## Graphical abstract

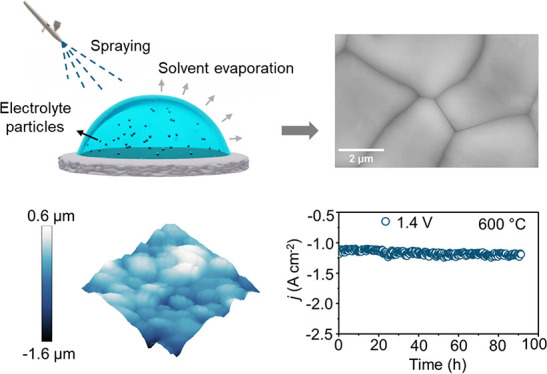

## 1 Introduction

The accelerating pace of climate change and the urgent need to decarbonize global energy systems demand scalable solutions for integrating intermittent renewable energy sources such as solar and wind into the grid. Effective large-scale energy storage technologies are essential to balance supply and demand, stabilize power networks, and enable the deep penetration of renewables. Reversible solid oxide-based electrochemical systems have emerged as attractive candidates for this purpose, offering the ability to operate in both fuel cell mode for electricity generation and electrolysis mode for renewable hydrogen production.^1^ Among these, protonic ceramic electrochemical cells (PCECs) are particularly promising due to their high efficiency, fuel flexibility, and favorable operating temperatures (400–600 °C), which enable rapid electrode kinetics and reduced thermal stress compared with conventional high-temperature systems. Their capability to efficiently convert and store energy in the form of hydrogen makes PCECs a viable pathway toward a sustainable, low-carbon energy infrastructure.^2–4^

The electrolyte is a critical component in PCECs, as it governs proton transport, contributes to the overall ohmic resistance, and directly influences cell durability. State-of-the-art proton-conducting electrolytes, such as barium cerate–zirconate–yttrium–ytterbium compositions (e.g., BaCe_0.7_Zr_0.1_Y_0.1_Yb_0.1_O_3−δ_, BCZYYb), exhibit high proton conductivity and low activation energy in the intermediate temperature range.^5^ Over the past decade, advances in BCZYYb processing and cell design have enabled promising electrochemical performance at reduced operating temperatures, opening the door to the use of low-cost metallic interconnects and other stack components that can significantly reduce manufacturing costs while maintaining long-term stability. Despite these advances, the practical implementation is still limited by fabrication challenges, including poor sinterability, thermal expansion mismatch with electrodes, and microstructural defects such as grain boundary porosity, which can disrupt proton transport pathways, weaken interfacial adhesion, and accelerate performance degradation.^6–8^ Addressing these issues requires dense, uniform electrolyte layers with strong electrode interfaces to minimize ohmic losses, stabilize the triple-phase boundaries (TPBs), and sustain high electrochemical performance over extended cycling.^9^

Various advanced deposition techniques, such as atomic layer deposition, chemical vapor deposition, and pulsed laser deposition, have been explored to fabricate electrolyte membranes.^10–13^ However, limited scalability and complexity make these vacuum-based methods impractical for large-scale PCEC manufacturing, underscoring the need for low-cost, scalable fabrication routes for dense electrolytes.^13^ Several nonvacuum fabrication techniques have been explored for producing dense BCZYYb electrolyte membranes, including inkjet printing, tape casting, and spray-based deposition.^14–17^ Among them, spray deposition methods, particularly when integrated with ultrasonication, have attracted attention for their ability to disperse particles uniformly and form thin, continuous electrolyte layers.^18^ For example, Taillades et al. optimized nozzle-substrate distance and spray velocity to produce crack-free electrolytes on 40 mm substrates, while Feng et al. examined how solid loading and the number of spray passes in wet powder spraying (WPS) influence particle arrangement prior to sintering, with deviations from optimal parameters leading to lose surface microstructures.^16,19^ In advanced WPS, a suspension of a solvent and electrolyte powder is atomized onto a substrate using ultrasonic energy; however, the requirement to finely control nozzle height limits flexibility for coating curved or irregular surfaces and constrains scalability.^20^

In this regard, traditional spray coating (TSC) offers a simpler, more adaptable, and cost-effective alternative for fabricating thin films. This process is widely employed due to its capability to enable cost-effective thin-film deposition over large and irregular surfaces. During the deposition of the thin-film slurry, the solvent rapidly evaporates, preventing the subsequent layer of the thin film from dissolving the previously deposited electrolyte layer. Owing to its simplicity and scalability, this traditional technique has also been adopted for semiconductor-based electronic device fabrication. It is regarded as one of the most effective methods for quasi-2D semiconductor ink deposition.^20–23^ However, limited studies have been reported on the fabrication of R-PCEC devices utilizing the TSC process. Shi et al. fabricated a thin electrolyte layer of hydrothermally prepared SDC electrolyte powder that showed the basic performances.^24^ Carpanese et al. demonstrated a dense 10 μm BaCe_0.85_Y_0.15_O_2.925_ electrolyte fabricated via traditional spraying, highlighting the importance of suspension formulation and deposition/sintering conditions in achieving high density.^25^ Nevertheless, that work required postdeposition isostatic pressing, indicating that further process optimization is needed to achieve dense, defect-free membranes without additional costly steps. Moreover, the persistent presence of porosity and small grain size have been identified as a major factor limiting the overall performance of the device based on the TSC process.^18^ Further, no prior studies reported long-term stability of higher performance and scalability of the fabricated dense BCZYYb electrolyte via TSC.

Although multiple fabrication processes can be employed, the proton conductivity in R-PCECs is ultimately dictated by achieving a compact, pore-free electrolyte microstructure.^26–28^ The densification of the electrolyte layer directly influences the ohmic resistance (*R*_o_), as morphological def**e**cts such as voids at grain boundaries can impede proton (H^+^) transport and degrade overall cell conductivity.^29,30^ Poorly bonded interfaces between the electrolyte and electrodes further hinder reaction kinetics, reducing efficiency, durability, and reversibility.^31–33^ In contrast, dense grain boundaries promote strong interfacial adhesion and faster electrochemical reactions, enhancing the long-term stability. In WPS, the initial particle arrangement, governed by solution viscosity, solvent volatility, dispersion, and evaporation dynamics, has a strong impact on densification.^19,25,34,35^ These parameters determine the particle packing density and the thin-film growth mode: (a) Volmer–Weber (3D island growth), (b) Stranski–Krastanov (mixed growth), or (c) Frank–van der Merwe (layer-by-layer 2D growth). Uniform 2D growth facilitates effective neck formation and grain boundary development during sintering, producing dense membranes. By contrast, the other growth modes often yield incomplete sintering and microporosity due to increase number of grain boundaries near the pore.^36,37^ Consequently, achieving highly dense, well-bonded spray-coated films requires precise control over the solution formulation to regulate the particle flow, packing, and deposition uniformity. Therefore, there is the necessity of fine-tuning electrolyte slurry formulation for the scalable fabrication of R-PCECs to achieve larger average grain size and a defect-free electrolyte layer for better performance and stability.

Here, we employed a cost-effective and scalable spray-coating approach to deposit a thin electrolyte film for the R-PCEC fabrication. By optimizing solvent composition and solute loading, we achieved a well-dispersed suspension that promoted uniform grain packing and high densification after sintering. The observed homogeneous grain compaction indicates a balance between coffee-ring and Marangoni flow effects during deposition. This refined microstructure directly translated into improved electrochemical performance, delivering a current density of 1.31 A cm^–2^ at thermoneutral voltage (1.3 V) in EC mode. The enhancement originates from increased bulk H^+^ conductivity and a stable electrolyte-electrode interface, enabling efficient proton transport across the triple-phase boundary. Cross-sectional FESEM-EDS analysis of the reduced full cell confirmed that the interface remained intact after extended operation, underscoring the durability of the fabricated membrane.

## 2 Experimental Section

### 2.1 Material Synthesis

The BCZYYb electrolyte powder was synthesized by following a solid-state reaction (SSR) process. The chemicals used in this process were barium carbonate (BaCO_3_), cerium oxide (CeO_2_), zirconium oxide (Zr_2_O_3_), yttrium oxide (Y_2_O_3_), and ytterbium oxide (Yb_2_O_3_), with high purity (99.9%) from Thermo Scientific. These precursors with BCZYYb stoichiometry were dissolved in ethanol, and a fine powder of electrolyte was obtained by using a double cycle of ball milling and calcination. The one cycle of this process involves ball milling for 24 h at 350 rpm in a planetary ball mill (PMV1-1L by MSE Supplies LLC), followed by drying the ball-milled solution at 120 °C in an oven and then calcination of the dried powder at 1100 °C for 10 h.

The synthesis of positive and negative electrode powders was carried out meticulously using a sol–gel process (Experimental details S1 in the Supporting Information). For this study, NiO-BaCe_0.7_Zr_0.7_Y_0.1_Yb_0.1_ and PrNi_0.7_Co_0.3_O_3−δ_ (PNC) were used as fuel and air electrode, respectively.

### 2.2 Formulation of Spray-Coating Slurry

The dispersive solution was prepared by mixing ethyl cellulose (EC) as thin-film formation aid, polyvinyl butyl (PVB) (Butvar B-98) as a binder, which was obtained from Thermo Scientific, and polyethylene glycol 200 (PEG) (Sigma-Aldrich) as a plasticizer in a solution containing isopropanol (MaxTite 99.9%) and α-terpineol (Thermo Scientific, 96%). For the variation in solvent composition, the wt % values of EC, PVB, and PEG were kept constant at 1, 1, and 2 wt %, respectively, in the total solution. To investigate solvent sensitivity, three dispersive solvent systems were prepared: (a) BCZYYb20A, which consisted of 76 wt % of isopropanol, (b) BCZYYb20B, which included 76 wt % of α-terpineol, and (c) BCZYYb20C, which was prepared by using an equal proportion of isopropanol (38 wt %) and α-terpineol (38 wt %) ensuring the maximum variation did not exceed ± 0.2 wt %. Similarly, the solution was heated and stirred at 60 °C for 1 h on a hot plate to obtain a homogeneous dispersive solvent. Next, electrolyte powder was added to the dispersive solvent, maintaining a 1:4 ratio, and the mixture was ball-milled for 24 h at 300 rpm. To ensure the proper dispersion of the electrolyte powder in the dispersive solvent, 1 wt % fish oil (Tape Casting Warehouse, USA) was added as a dispersant to the total solution before ball milling the electrolyte powder in the ball mill. The optimization of the solution was achieved by varying the composition of the dispersive solvent and the ratio of the electrolyte powder in the solution.

### 2.3 Fabrication of Full Cells

First, electrolyte layer-supported pellets were fabricated using a 12 mm die. The two layers, containing the anode and the anode functional layer, were pressed together by applying 200 MPa to a hydraulic press machine. These pellets were presintered at 920 °C for 2 h to gain mechanical strength before spraying the electrolyte layer on the substrate. The presintered anode pellets were placed on a hot plate at 80 °C to maintain their temperature. The ball-milled electrolyte solution was sprayed on the substrate using an airbrush (Master Airbrush G233-SET) with a 0.3 mm nozzle hole. The airbrush was connected to a single cylinder piston compressor pump (AS18K-2, Ningbo Haosheng Pneumatic Machinery), and the applied pressure was maintained at ∼1 atm by regulating the pressure gauge connected to the pump (Figure S1). The fabricated half cells were further presintered at 920 °C for 3 h to burn out the organic compounds present in the electrolyte layer and to achieve a uniform distribution of electrolyte powder particles on the substrate surface. These presintered half cells were cosintered in a large muffle furnace for 10 h at 1425 °C.

The electrochemical analysis was performed by screen printing a cathode layer. For screen printing, two separate solutions were prepared by adding 5 wt % V-006 (Heraeus) and 20 wt % Solsperse 28000 (Lubrizol) in α-terpineol separately and stirring until complete dissolution. Thus, the PNC ink was prepared using PNC powder in the two solutions mentioned above and painted onto the active electrode surface area of 0.178 cm^2^ on the obtained half cells from the spray-coating process. This was further calcined at 1050 °C for 5 h to evaporate the solvents present in the electrode slurry.

### 2.4 Compositional and Morphological Characterization

The pristine perovskite structure of the BCZYYb electrolyte powder was analyzed with the help of X-ray diffraction (XRD, Rigaku SmartLab) in the range of 20–80 (2θ) using a rotating Cu anode source. For the morphological study of half cells as well as reduced full cells, SEM (Scanning Electron Microscopy, TFS Quattro S) integrated with energy-dispersive X-ray spectroscopy (EDS) was used in two different modes, i.e., backscattered and secondary electron modes. The grain size distribution analysis was done repetitively three times on obtained SEM images of the electrolyte surface following the intercept method. Furthermore, color-SEM was performed for line-scanning analysis of the electrolyte surface, and atomic force microscopy (AFM, Park NX10 AFM) was used to characterize the surface roughness by scanning the area of 20 μm × 20 μm for each half cell. The mean square roughness measurement was done by using XEI V-5.1.6 software published by Park Systems. EDS characterization was performed for elemental mapping at the cross-section of the reduced full cell.

### 2.5 Electrochemical Performance Investigation

Initially, a silver grid and a silver paste were applied to cover both sides of the electrodes in the fabricated full cell. The cell assembly was subsequently affixed to a lab-designed alumina tube testing fixture using a ceramic sealant (Ceramabond 552) to ensure a gastight configuration. For fuel cell mode operation, the fuel electrode was supplied with hydrogen gas, starting at a flow rate of 3 sccm and gradually increasing to 20 sccm while maintaining the operation temperature at 600 °C. Once the OCV exceeded 1.0 V, comprehensive FC mode testing was performed. A Parstat MC (potentiostat from Princeton Applied Research) was used to perform all electrochemical assessments and electrochemical impedance spectroscopy (EIS) characterization, and VersaStudio software (AMETEK Scientific Instruments) was used to record the test data. For electrolysis cell mode operation, the air electrode was initially supplied with oxygen at a relative humidity of 20%, and the oxygen flow rate to the air electrode was maintained at 40 sccm. The ohmic and polarization resistance of the fabricated full cell were measured within a 10^6^ to 10^–1^ Hz frequency range at OCV in FC mode and at 1.3 V in EC mode on applying an amplitude voltage of 30 mV. The ramping and cooling rates were kept at 2 °C min^–1^.

## 3 Results and Discussion

### 3.1 Effect of Solution Parameters on the Spray-Coated BCZYYb Electrolyte Microstructure

In the fabrication of PCECs, achieving a dense electrolyte film depends on two critical stages: the initial arrangement of particles in the green body and the subsequent sintering process. According to the Frank–van der Merwe growth model, closely packed particles in the green state create numerous contact points between neighbors, which enhance mass transport during sintering. During densification, the pore coordination number (*P*_c_) decreases as necks form between particles, facilitating pore elimination.^19,28^ In the final sintering stage, grain growth is influenced by Ostwald ripening, and pore shrinkage can be described by eq 1:^38^

$$\gamma_{\mathrm{bg}}=2\gamma_{s}\cos\frac{\varphi }{2}$$ 1

where γ_bg_ is known as intergranular boundary energy or surface energy, γ_s_ is surface tension or free boundary energy, and φ is the dihedral angle. For the formation of compact grain boundaries, φ must be high enough for the increment of the threshold coordination number and the pore coordination number below the critical value of threshold coordination number. Thus, precise control of particle arrangement before sintering is essential for promoting efficient pore elimination and uniform grain growth, ultimately yielding a mechanically robust, dense, and highly conductive electrolyte microstructure.^19,38–40^

In spray-based deposition methods, including wet powder spraying, the final particle arrangement depends on four key preliminary steps: droplet uniformity and transport, wettability upon impact, spreading, and drying.^19^ In this work, we optimized the spray-coating slurry by fixing the binder, dispersant, and BCZYYb electrolyte powder while varying the solvent composition, specifically the ratio of isopropanol to α-terpineol (Figure 1a). Uniform particle suspension ensures consistent droplet size, which in turn leads to even particle distribution on the substrate. As droplets travel toward the substrate, the volatility and viscosity of the solvent govern the rearrangement of particles within the growing film. Upon impact, controlled wetting and spreading help avoid particle clustering, and the interaction between droplets is strongly influenced by surface tension gradients. The Marangoni flow phenomenon where fluid moves from regions of low surface tension to high surface tension can redistribute particles and counteract uneven settlement.^34,41^

**Figure 1:**
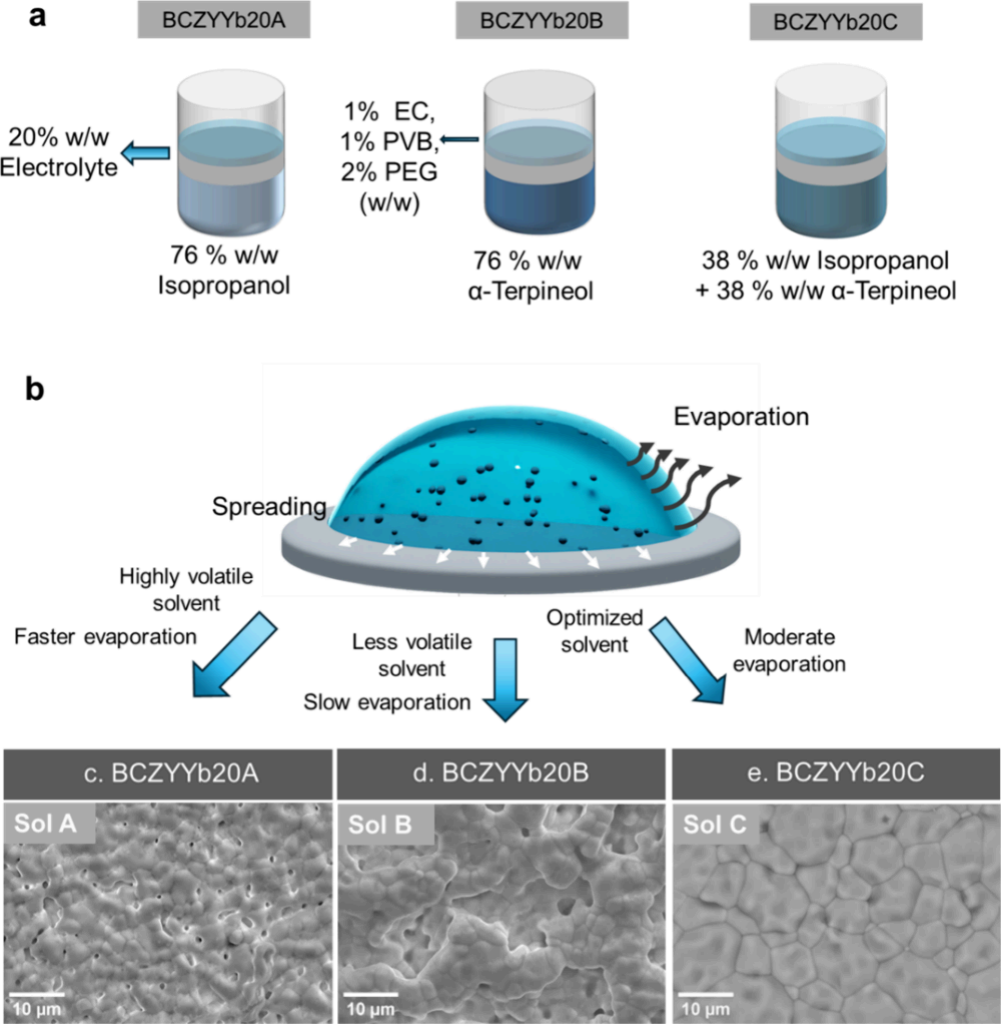
Fabrication process of the electrolyte layer via a WPS process and its impact parameters. (a) Slurry optimization through tuning solvent composition. (b) Schematic illustration of key factors influencing drying behavior. SEM images of electrolyte surfaces prepared with different slurry formulations: BCZYYb20A, with high volatile content, exhibits rapid solvent evaporation and pore formation; BCZYYb20B, with reduced volatile content, shows organic particle aggregation; and BCZYYb20C, with balanced volatile and organic solvent content, achieves a dense microstructure with compact grain boundaries.

During drying, the dynamics of solvent evaporation becomes decisive (Figure 1b). Both overly rapid and excessively slow evaporation can induce the coffee-ring effect, leading to nonuniform deposition via 3D island growth or mixed growth modes.^34,42^ In our experiments, BCZYYb20A (76 wt % isopropanol) evaporated rapidly, resulting in the formation of micropores and nonuniform surfaces (Figure 1c). In contrast, BCZYYb20B (76 wt % α-terpineol) evaporated slowly, leading to particle accumulation and irregular packing (Figure 1d). The optimized BCZYYb20C formulation, with a balanced 38 wt % isopropanol and 38 wt % α-terpineol, produced the most compact grain boundaries (Figure 1e). This balance promoted controlled leveling and an inward Marangoni-driven flow that counteracted coffee-ring effects, enabling uniform 2D film growth and higher densification.^43^

### 3.2 Morphology and Grain Structure of Spray-Coated Electrolytes

The BCZYYb powder was synthesized via the SSR route and used to prepare the spraying solutions. XRD characterization confirmed the phase purity of the synthesized powder (Figure S2). Figure 2a shows the evolution of electrolyte microstructures as the solute loading (% w/w) in the solution was varied. At low solute loading (10% w/w, BCZYYb10C), a mixed effect of the BCZYYb20A and BCZYYb20B surfaces was observed, which can be attributed to nonuniform particle arrangement prior to sintering and the presence of the coffee-ring effect, leading to increased pore coordination.^44^ Using the optimized solvent formulation (BCZYYb20C, 20% w/w), we obtained a pore-free and compact electrolyte surface was obtained. Increasing the solute loading further to 30% w/w (BCZYYb30C) caused visible rifts between grain boundaries. These observations indicate that particle distribution and arrangement during spraying play critical roles in reducing porosity and enhancing grain compaction.

**Figure 2:**
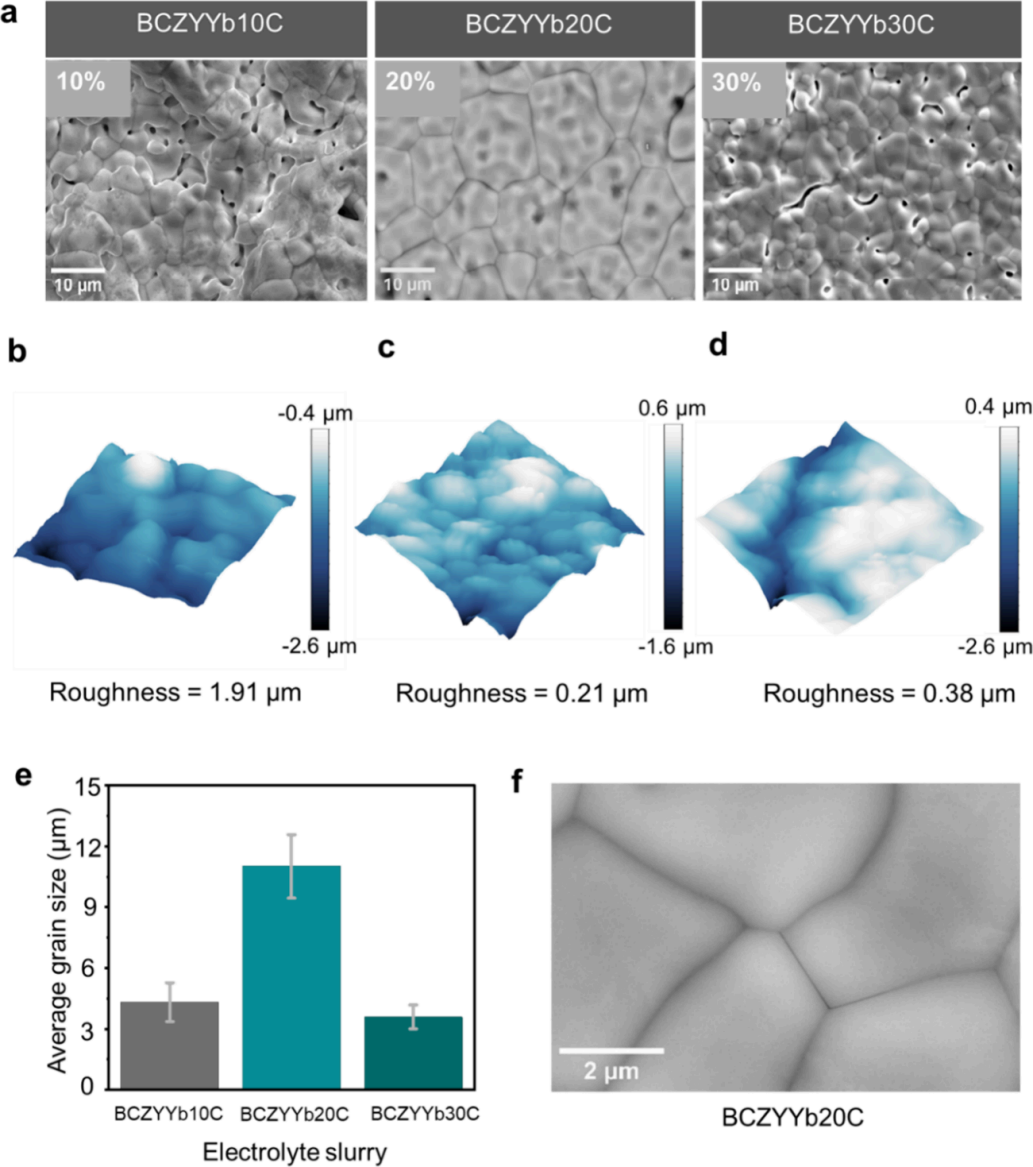
Influence of solute loading (% w/w) in the spraying slurry on BCZYYb electrolyte grain growth. (a) SEM images of sintered half-cell microstructures with electrolyte loadings of 10, 20, and 30%. (b–d) AFM surface roughness maps of BCZYYb10C (b), BCZYYb20C (c), and BCZYYb30C (d). (e) Comparison of average surface grain size distributions in the fabricated half cells. (f) High-magnification SEM image of BCZYYb20C showing no elemental segregation at grain boundaries.

AFM characterization was performed to further examine the surface roughness and grain boundary growth. BCZYYb10C exhibited the highest roughness (∼1.91 μm) due to the presence of macropores (Figure 2b), whereas BCZYYb20C showed an average roughness of ∼0.21 μm (Figure 2c), comparable to tape-cast BCZYYb half cells reported by Bian et al.^45^ BCZYYb30C had an intermediate roughness (∼0.38 μm, Figure 2d), suggesting nonuniform grain growth along the *Z*-axis. These AFM observations are consistent with SEM imaging, confirming the correlation between the surface morphology and solute concentration. The influence of solute loading on the grain size was also evaluated (Figure 2e). Using the intercept method (ASTM E112), the average grain sizes were measured as ∼4.3, 11.0, and 3.5 μm for BCZYYb10C, BCZYYb20C, and BCZYYb30C, respectively, with coefficients of variation of 22, 14.2, and 16.3%.^46^ This indicates a more uniform grain distribution in BCZYYb20C, while BCZYYb10C and BCZYYb30C exhibited higher size variability. Reduced grain size in BCZYYb10C and BCZYYb30C can be attributed to pore formation from nonuniform particle packing prior to sintering.

High-magnification SEM of BCZYYb20C (Figure 2f) revealed a smooth surface with compact grains and no observable segregation or impurities at the grain boundaries. Line-scanning analysis along L1 and L2 confirmed a uniform elemental distribution, indicating that the optimized spray process effectively mitigated excessive cation diffusion, which could otherwise hinder electrolyte performance (Figure S3).

### 3.3 Electrochemical Performance of Full Cells with Optimized Electrolyte Layers

SEM characterization of the fabricated half cells revealed that BCZYYb20C and BCZYYb30C exhibited denser grain structures compared with other formulations. These two half cells were selected for full-cell fabrication by screen printing PrNi_0.7_Co_0.3_O_3‑δ_ (PNC) cathodes,^47^ and their electrochemical performance was evaluated in fuel cell (FC) mode at 600 °C. As shown in Figure 3a, BCZYYb20C achieved a higher peak power density (0.962 W cm^–2^) compared to BCZYYb30C (0.747 W cm^–2^), representing the highest reported performance by about 31% for BCZYYb7111 PCFCs prepared via wet powder spraying (WPS) (Figure 3b).^16,17,19,48^ For comparison, the peak power density of full cells fabricated via atmospheric plasma-spraying, WPS, spin coating, and tape casting fabrication were substantially lower (Tables S1 and S2).^6,24,26,32,49^

**Figure 3:**
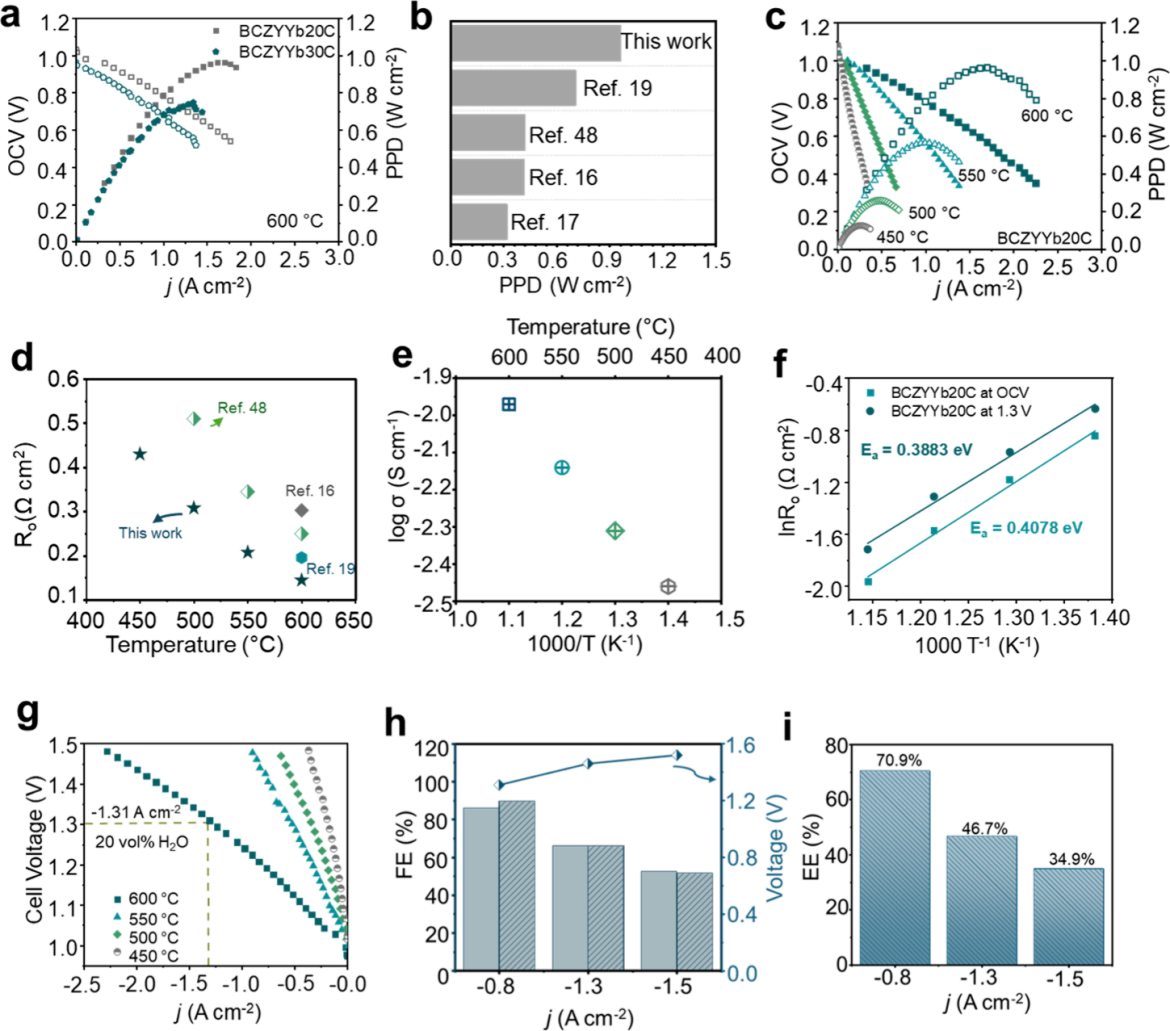
Correlative electrochemical performance of WPS-fabricated BCZYYb electrolytes. (a) *I*–*V*–*P* curves of BCZYYb20C and BCZYYB30C in FC mode at 600 °C, highlighting the effect of the electrolyte microstructure on cell performance. (b) Comparison of peak power density across WPS-fabricated samples. (c) *I*–*V*–*P* curves of BCZYYB20C in FC mode. (d) Arrhenius plot of ohmic resistance, revealing temperature-dependent transport behavior. (e) Total proton conductivity by fabricated electrolytes. (f) Activation energy extracted in both FC and EC modes. (g) *I*–*V* curves of BCZYYb20C in EC mode from 450 to 600 °C. (h) Faradaic efficiency (FE) was measured at −0.8, −1.3, and −1.5 A cm^–2^ in EC mode. The measurements for each were repeated twice with 20 vol % H_2_O carried by 40 sccm O_2_ to the oxygen electrode, and 20 sccm dry H_2_ gas was supplied to the fuel electrode at 600 °C. (i) Corresponding energy efficiency (EE) was evaluated under the same conditions.

The lower performance of BCZYYb30C can be attributed to its reduced open-circuit voltage (OCV = 0.95 V) and the presence of rifts between grain boundaries on the electrolyte surface. Electrochemical impedance spectroscopy (EIS) confirmed this observation, showing an increase in area-specific ohmic resistance (ASR_o_) to 0.16 Ω cm^2^ for BCZYYb30C compared to 0.14 Ω cm^2^ for BCZYYb20C (Figure S4b), highlighting the impact of microstructural defects on proton conduction. The temperature-dependent FC performance of BCZYYb20C with a PNC cathode is shown in Figure 3c, with pure H_2_ supplied to the fuel electrode. The high OCV value of 1.03 V at 600 °C indicates a compact, well-sealed electrolyte membrane. The maximum power densities at 600, 550, 500, and 450 °C were 962, 564, 258, and 126 mW cm^–2^, respectively, comparable to the powder-packed PNC electrode.^32^ EIS analysis further confirmed low electrolyte resistance, with *R*_o_ values of 0.14, 0.21, 0.31, and 0.43 Ω cm^2^ at 600–450 °C, respectively (Figure 3d). Corresponding proton conductivities were in the 10^–2^ S cm^–1^ range at 600 °C in FC mode, decreasing with the temperature (Figure 3e), and the activation energy was correspondingly low (Figure 3f).

The electrochemical performance of BCZYYb20C was also evaluated in electrolysis (EC) mode. The cell generated current densities of −0.19, −0.34, −0.52, and −1.31 A cm^–2^ at 450, 500, 550, and 600 °C, respectively, under 1.3 V (Figure 3g), with dry H_2_ at the fuel electrode and 20% humidified air at the cathode. The compact, dense electrolyte layer of BCZYYb20C is supported by the low ASR_o_ values (Figure S5), indicating that proton conduction dominates and facilitates efficient ion transport. Stepwise current testing at −0.8, −1.3, and −1.5 A cm^–2^ at 600 °C further confirmed the stable voltage generation (Figure S6). Faradaic efficiency (FE%) and energy efficiency (EE%) were also evaluated to assess electrochemical conversion performance. BCZYYb20C achieved an FE of 89.7% at −0.8 A cm^–2^, decreasing with higher current densities, while EE reached 70.9% at −0.8 A cm^–2^, demonstrating efficient conversion of electrical energy into chemical energy for large-scale hydrogen production target for R-PCEC (Figures 3h,i). These results underscore the critical role of the optimized electrolyte microstructure in achieving high electrochemical performance and efficient proton transport.

### 3.4 Thermal and Electrochemical Stability of Full Cells under Dynamic and Long-Term Operation

The BCZYYb20C full cell underwent repeated heating and cooling operations during normal operation. To evaluate its performance under thermal cycling, the cell was subjected to temperature variations between 600 and 500 °C. At each target temperature, *I*–*V* curves were recorded, and current density at 0.2 V was extracted (Figure S7a,b). EIS measurements confirmed the *I*–*V* observations (Figure S7c,d). As shown in Figure 4a, the current density at the OCV remained stable over five cycles, demonstrating the robustness of the cell. The recovery of performance at high temperature during back-and-forth cycling can be attributed to enhanced proton and oxide ion transport due to thermal activation. Thermal energy promotes the mobility of charge carriers, improving transport at the electrode/electrolyte interfaces.^32,47^

**Figure 4:**
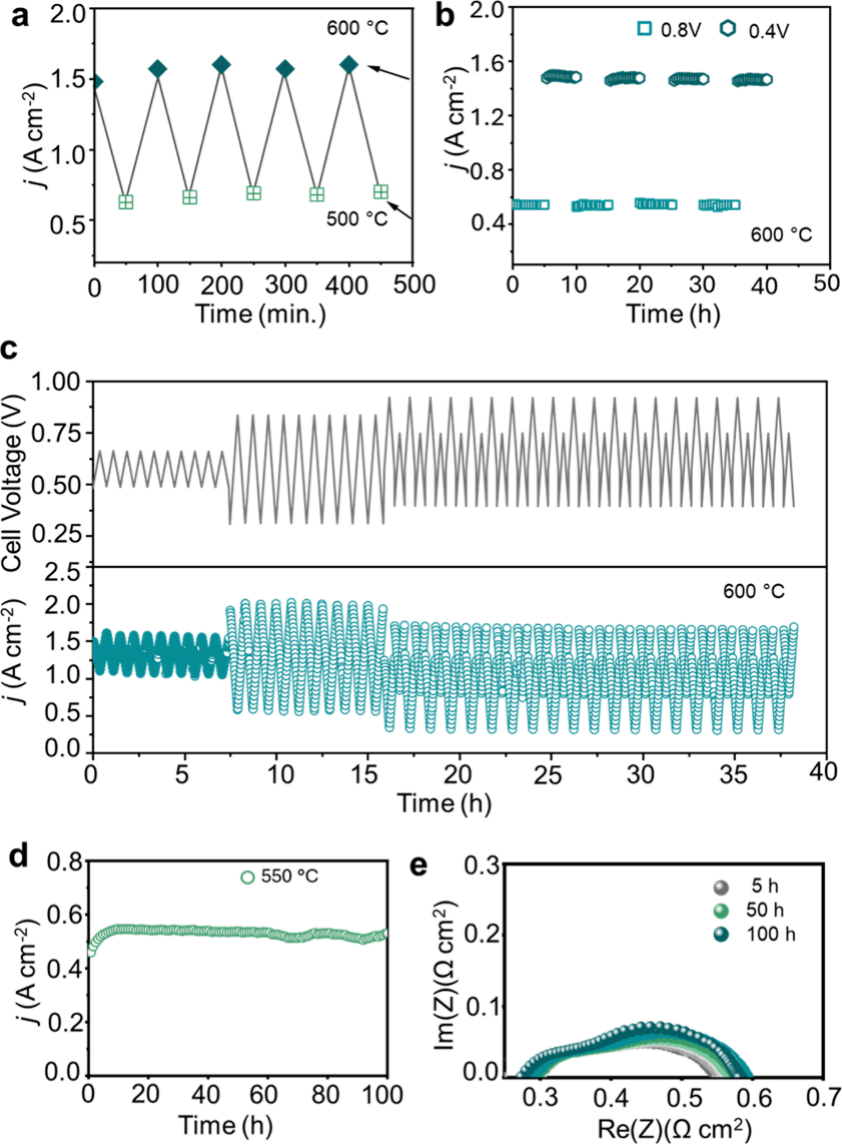
Comprehensive durability assessment under extreme operational conditions in FC mode. (a) Thermal shock recovery evaluation of BCZYYb20C between 600 and 500 °C over five cycles, demonstrating stable proton conduction and fuel cell performance. (b) Static current response under constant voltages of 0.4 and 0.8 V at 600 °C, each maintained for 5 h over five cycles. (c) Extended performance under cyclic voltage operation with step changes: (i) 0.4 V/0.6 V, (ii) 0.2 V/0.8 V, (iii) 0.5 V/0.9 V, and (iv) 0.3 V/0.7 V at 600 °C. (d) Long-term stability at 0.7 V at 550 °C over 100 h. (e) EIS spectra collected during the 100 h stability test at 550 °C.

To further prove stability under varying voltage conditions, stepwise voltage tests were conducted. The applied cell voltage was varied from 0.8 to 0.4 V, with each voltage held for 5 h (Figure 4b). This procedure was repeated for a total of five cycles, and EIS measurements were performed before and after the test (Figure S8a). The results indicate that the cell can withstand variations in applied voltage with strong resilience at the electrolyte/electrode interfaces. Notably, the polarization resistance (*R*_p_) decreased after testing, suggesting that interfacial chemical bonding remained stable.^1^

The interface stability and material integrity were further investigated through elongated transient tests. The cell potential was cycled between 0.2 and 0.9 V over 10 cycles, using sequences of (i) 0.4/0.6 V, (ii) 0.2/0.8 V, (iii) 0.5/0.9 V, and (iv) 0.3/0.7 V (Figure 4c). The current density showed minimal deviation across these cycles, indicating stable interfaces and compact grain boundaries that prevent electronic leakage. EIS characterization during these tests revealed that the ohmic resistance remained nearly constant before and after cycling (Figure S8b), confirming the ability of the cell to endure dynamic electrochemical conditions. Long-term stability tests were conducted at 550 °C for 100 h. Figure 4d shows that the current density initially increased from 0.45 to 0.54 A cm^–2^ within the first 10 h and then remained stable, with a degradation rate of only 0.02% h^–1^. EIS analysis during the test revealed a decrease in *R*_o_, confirming the robustness of the cell even at lower operating temperatures (Figure 4e).

The durability of BCZYYb20C in the EC mode was also evaluated. Stepwise voltage tests were performed at 1.4, 1.3, and 1.2 V, holding each voltage for 30 min per cycle. In the first cycle at 600 °C, the current densities were −1.37, −1.01, and −0.66 A cm^–2^ at 1.4, 1.3, and 1.2 V, respectively, and these values remained nearly constant over nine cycles, with an overall degradation of 4.5% (Figure 5a and Figure S9a). Transient voltage cycling tests between 1.1 and 1.5 V over 20 cycles further confirmed the stability of the electrolyte/electrode interfaces. The results were supported by decreases in both ASR_o_ and polarization resistance (ASR_p_), indicating improved hydrogen evolution reaction kinetics and strong interfacial contact (Figure 5b and Figure S9b). Additional dynamic testing was conducted by varying cell voltages among 1.2/1.3, 1.1/1.4, and back to 1.2/1.3 V over 10 cycles for each transition. The current density showed a slight increase, attributed to enhanced ohmic and polarization resistances, further supporting the interface integrity (Figure 5c and Figure S9c). Reversible operation was evaluated by modulating the applied voltage between 1.3 and 0.8 V over 10 cycles. The current density responded immediately to the applied voltage, indicating stable interfacial adhesion and rapid proton transport (Figure 5d and Figure S10a).

**Figure 5:**
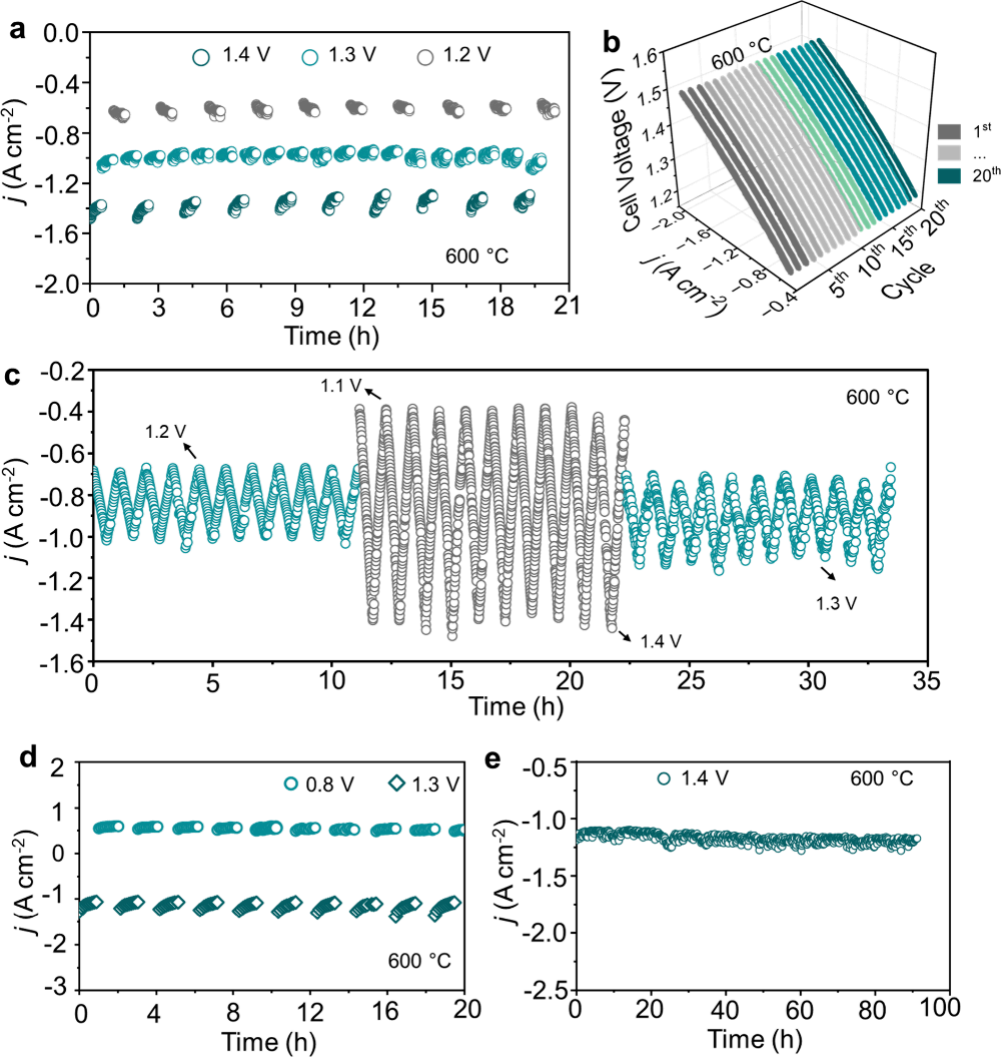
Structural and electrochemical durability assessment of BCZYYb20C in EC mode. (a) Extended current density evaluation by applying stepwise voltages of 1.4, 1.3, and 1.2 V, with 20% H_2_O + 40 sccm O_2_ at the oxygen electrode and 20 sccm H_2_ at the hydrogen electrode, at 600 °C. (b) Cyclic voltammetry from 1.1 to 1.5 V for 20 cycles, demonstrating stable performance. (c) Dynamic current response under repeated voltage changes (1.3 to 1.2, 1.1 to 1.4, and 1.3 to 1.2 V) for 10 cycles each. (d) Reversible cyclic assessment between 1.3 and 0.8 V. (e) Long-term stability under 20% humidity at 1.4 V for 100 h.

Finally, long-term EC testing at 1.4 V and 600 °C for 100 h demonstrated minimal fluctuation in the current density, with only a 0.01% increase, confirming excellent electrochemical robustness (Figure 5e). EIS measurements before and after the 100 h test showed significant decreases in both *R*_o_ and *R*_p_, indicating seamless interfaces and maintenance of structural integrity during prolonged operation (Figure S10b). Overall, these results demonstrate that the BCZYYb20C electrolyte layer maintains a compact, dense microstructure and strong adhesion to electrodes, ensuring outstanding thermal and electrochemical stability under dynamic and long-term operation. Further, the results obtained in this study and the experiments conducted demonstrated reproducibility (Table S3).

### 3.5 Post-Test Structural and Interfacial Integrity Analysis

The BCZYYb20C full cell demonstrated remarkable electrochemical performance and durability in both the FC and EC modes. Electrochemical characterization indicated enhanced interfacial contact with the electrodes and improved the reaction kinetics. Surface characterization of the BCZYYb20C electrolyte further confirmed a compact layer with a uniform elemental distribution.

To validate structural integrity after prolonged testing, post-test cross-sectional analysis was conducted. Figure 6a shows a dense, 15 μm-thick electrolyte layer and stable interfaces among the air electrode, electrolyte, and AFL. Higher-magnification cross-sectional images revealed that both the air electrode/electrolyte and electrolyte/AFL interfaces remained intact even after extended operation (Figure 6b,c), consistent with reported observations.^31^ These results confirm the robustness and stability of the electrode/electrolyte interfaces under prolonged electrochemical cycling. Additionally, a comprehensive EDS analysis was performed to evaluate elemental diffusion at the electrolyte/AFL interface, with particular attention to cation migration (Figure 6d).

**Figure 6:**
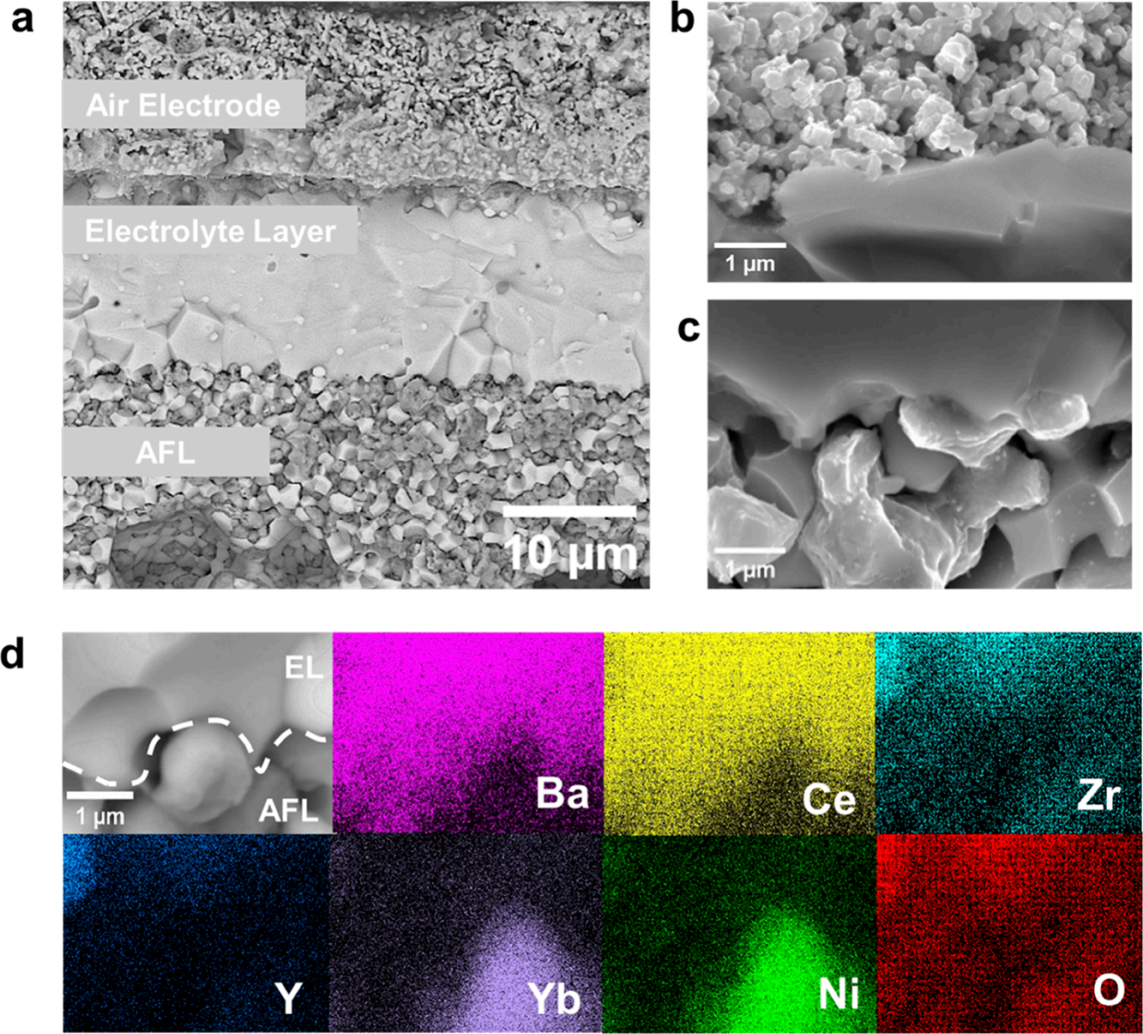
Interfacial integrity and structural characteristics of reduced cells after testing. (a) Cross-section characterization of the cell exposing interfacial bonding and reduction of NiO present in the anode into Ni that facilitates the conduction of protons. (b, c) SEM images of electrode/electrolyte interfaces in the post-test cell. (d) EDS elemental mapping at the EL/AFL interface to visualize spatial distribution and possible interdiffusion of constituent elements.

Color-SEM characterization further confirmed minimal diffusion at the air electrode/electrolyte interface, as shown in Figure S11, indicating that the fabrication process effectively mitigates detrimental interfacial cation movement. Therefore, these post-test analyses demonstrate that the BCZYYb20C electrolyte layer maintains structural integrity and stable interfaces, supporting sustained high performance in both fuel cell and electrolysis operations.

### 3.6 Scaling Up of the Spray-Coating Process

The optimized slurry composition of solution C with a solute loading of 20% w/w produced a dense, pore-free electrolyte microstructure with uniform grains. Initially, this spray-coating solution was used to fabricate 1.2 cm diameter half cells, serving as a baseline to validate the efficacy of the proposed formulation and deposition process. The successful fabrication and performance of these button-sized cells confirmed the reliability of the approach.

Encouraged by these results, the process was extended to a larger 2.5 cm diameter anode substrate to evaluate scalability (Figure S12). The resulting large-area half cell demonstrated only 21% shrinkage after sintering (Figure 7a), reflecting high densification and consistent microstructural quality across an increased area (Figure 7b). This outcome highlights the potential of the optimized spray-coating solution for producing uniform, high-quality electrolyte layers on larger scales.

**Figure 7:**
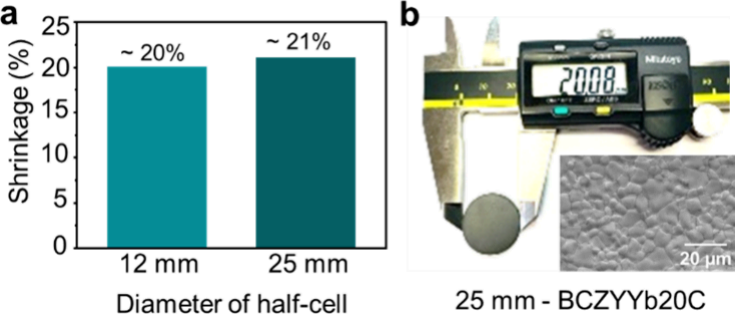
Fabrication of a defect-free electrolyte film on a 25 mm diameter anode substrate. (a) The half cell achieved 21% linear shrinkage after sintering, comparable to that of small button cells, indicating similar sinterability across different sizes. (b) Pronounced shrinkage of a 25 mm BCZYYb20C half cell to 20 mm after sintering at 1425 °C, without any warping or cracking, and the SEM image of the electrolyte surface showing a dense, defect-free microstructure, demonstrating the potential of the WPS method for scalable fabrication.

Beyond achieving dense microstructures, this approach offers several practical advantages for industrial-scale production. Unlike ultrahigh vacuum-based methods, the nonvacuum spray-coating process is low-cost, simple to implement, and compatible with complex or large geometries. The ability to tune solvent composition and solute loading enables precise control over particle packing and film uniformity, providing flexibility for different substrate sizes and shapes. Collectively, these features suggest that the proposed methodology can be readily adapted for mass production of protonic ceramic electrochemical cells, offering a feasible pathway toward cost-effective, scalable fabrication while maintaining the structural and electrochemical integrity of the electrolyte layer.

## 4 Conclusions

In this work, an optimized spray-coating process was developed for fabricating BCZYYb electrolyte layers, emphasizing the slurry composition and solute loading to achieve dense, pore-free microstructures. Surface characterization confirmed a uniform grain size and compactness, demonstrating that controlled particle arrangement during deposition is critical for achieving high-quality electrolytes. The final microstructure was influenced by both the coffee-ring effect and Marangoni flow with controlled droplet spreading and solvent evaporation promoting uniform 2D particle growth. Sintering of homogeneously distributed particles resulted in compact grains with minimal pores, ensuring efficient proton transport and avoiding insulating features at grain boundaries. SEM and EDS analyses confirmed the absence of elemental segregation or precipitation, validating the effectiveness of the optimized spray-coating approach. Electrochemical testing of full cells demonstrated high performance, achieving a peak power density of 0.96 W cm^–2^ in FC mode and a current density of 1.31 A cm^–2^ at 1.3 V in EC mode at 600 °C. The cell also exhibited excellent stability, maintaining performance over 100 h at 550 °C with minimal degradation. Moreover, the approach was successfully scaled to a 25 mm diameter anode substrate, showing consistent densification, dimensional stability, and defect-free surfaces, highlighting its potential for large-scale, cost-effective production. Future efforts will focus on further enhancing long-term stability and proton conductivity as well as advancing toward stack-level fabrication and integration with metallic interconnects for practical energy conversion applications. In summary, the optimized slurry formulation combined with a conventional spray-coating process provides a reproducible and scalable route toward MW-scale hydrogen generation modules.

## Supplementary Material



## Data Availability

The data that support the findings of this study are available from the corresponding author upon the reasonable request.

## Notes

Synthesis of electrodes, schematic diagram of traditional spray-coating fabrication, XRD characterization, color-SEM characterization, performance comparison table, statistical reproducibility data of testing, extended galvanic stability performance, thermal cyclic *I*–*V* curve, EIS characterization corresponding to each electrochemical analysis, post-test color-SEM characterization, and visual dimension analysis of scalable 2.5 cm half cell (PDF)
