# Supplementary material for: Scalable Solution-Processed Electrolyte Membranes with Optimized Microstructure for High-Performance Protonic Ceramic Electrochemical Cells
Source: ACS Appl Mater Interfaces. 2025 Dec 1;17(50):67813–22. doi: 10.1021/acsami.5c16287 (PMC12723646; doi:10.1021/acsami.5c16287)
Supplement: Supplementary file 1 [file am5c16287_si_001.pdf]

SUPPORTING INFORMATION

Scalable Solution-Processed Electrolyte Membranes with Optimized Microstructure for High-Performance Protonic Ceramic Electrochemical Cells

Anshu Kumari[1], Shuanglin Zheng[1], Saroj Karki[1], Idris Temitope Bello[1], Jiufeng Ruan[1], Yuqi Geng[1], Hanping Ding[1,*]

[1] School of Aerospace and Mechanical Engineering, University of Oklahoma, Norman, OK 73019 USA.

[*] Corresponding author: hding@ou.edu

S1. Experimental details

Synthesis of Electrodes

Fuel electrode

For the synthesis of the fuel electrode, yttrium oxide (Y_2O_3), ytterbium oxide (Yb_2O_3), barium nitrate [$Ba(NO_3)_2$], zirconium dinitrate oxide hydrate [$ZrO(NO_3)_2 \cdot xH_2O$], cerium nitrate hexahydrate [$Ce(NO_3)_3 \cdot 6H_2O$], nickel nitrate hexahydrate [$Ni(NO_3)_2 \cdot 6H_2O$] were used as precursors whereas ethylenediaminetetraacetic acid (EDTA) and citric acid (CA) were used as accelerating agents for combustion. Metal salt precursors were added together with EDTA and CA in deionized (DI) water, and the mixture was stirred to obtain a homogeneous solution. Thermo Scientific provided all the chemicals in this study with a purity of 99.9%.

The nitrate solutions of Y_2O_3 and Yb_2O_3 were prepared separately by dissolving them in nitric acid and added to the metal salt solution. The pH of the solution was kept neutral by adding NH_4OH (ammonium hydroxide) solution. The resulting solution was maintained at 300 °C to induce auto-ignition. After combustion, the ashes were collected in a crucible and then calcined for five hours at approximately 1000 °C. The calcined powder was ground into a fine powder, maintaining a ratio of 3:2:1 for nickel oxide (NiO), electrolyte powder, and corn starch, respectively, to prepare the anode powder.

Air electrode

For air electrode, precursors used were praseodymium nitrate hydrate [$Pr(NO_3)_3 \cdot 6H_2O$], $Ni(NO_3)_2 \cdot 6H_2O$, and cobalt nitrate hexahydrate [$Co(NO_3)_2 \cdot 6H_2O$] (with 99.9% purity from Thermo Fischer). These precursors were dissolved along with EDTA and CA in the DI water to promote combustion. The ratio of the molar concentration of the metal cation to EDTA was maintained at 1:1.5:1. The prepared solution was kept at a constant temperature of 300 °C for three hours to facilitate gel formation. After three hours, the ashes were collected, followed by calcination for 5 hours at a temperature of 1000 °C.

S2. Supplementary Results

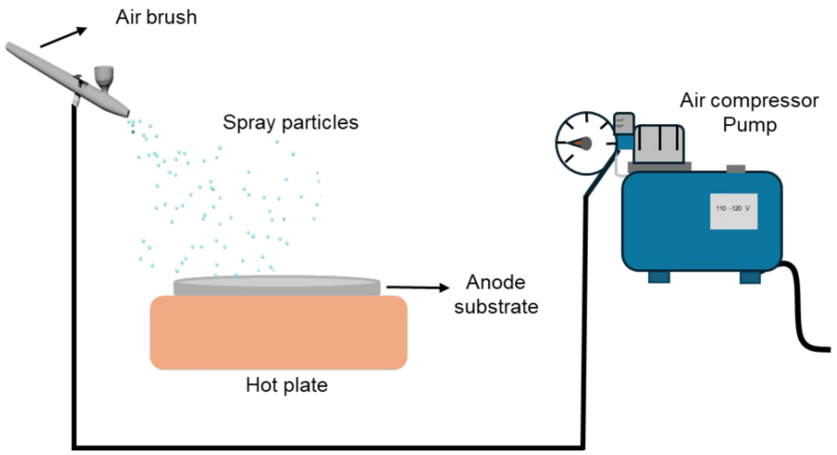

Figure S1. Schematic diagram of thin film fabrication of electrolyte on anode substrate via traditional spray coating (TSC) process.

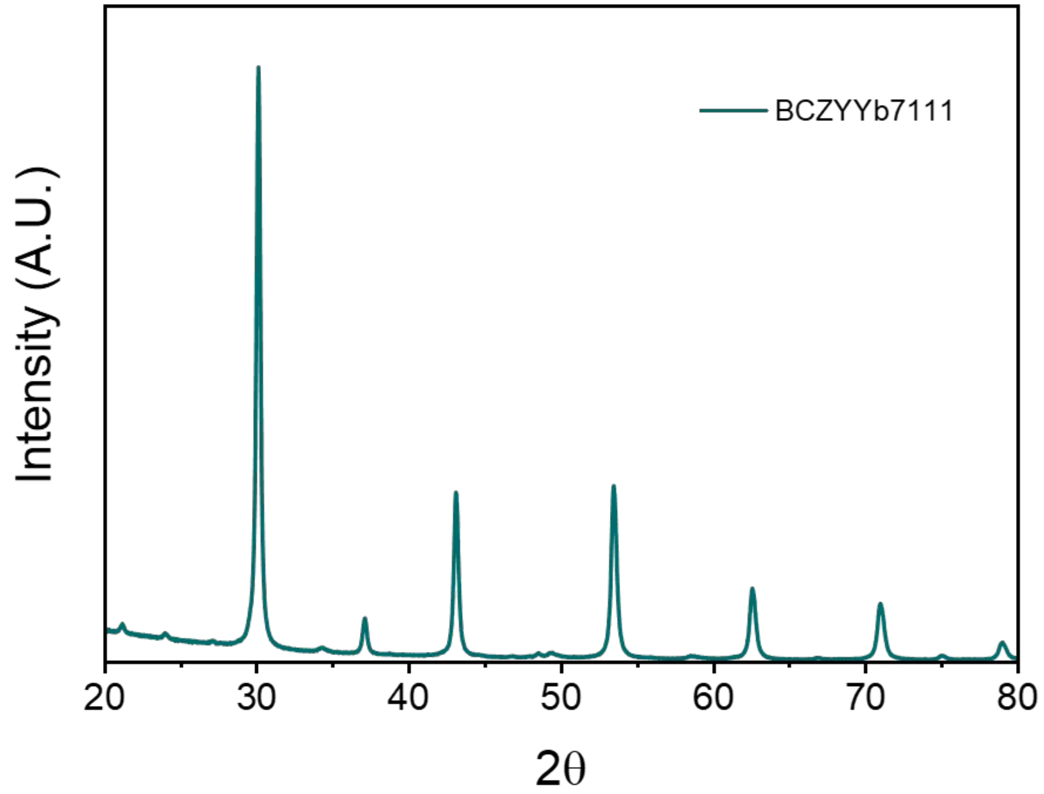

Figure S2. XRD characterization of synthesized $BaCe_{0.7}Zr_{0.1}Y_{0.1}Yb_{0.1}$ (BCZYYb7111) electrolyte powder from solid-state sintering process.

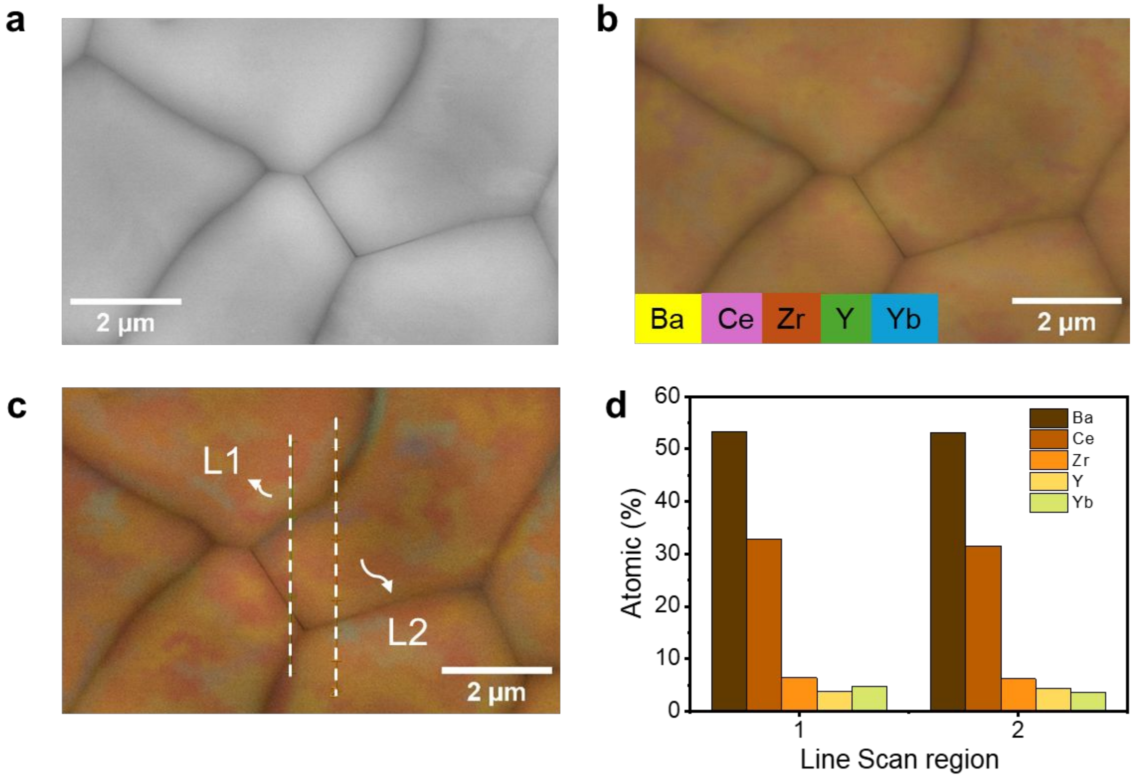

Figure S3. Color SEM characterization of BCZYYb20C: a) Absence of impurities at grain boundaries on higher magnification characterization. **b)** Uniform distribution of elements on the electrolyte surface. **c), d)** Uniform elemental distribution on line scanning at two different sites of BCZYYb20C electrolyte's surface.

Table S1. Comparison of the obtained peak power density with the reported literatures.

Fabrication Process	Electrolyte| air electrode	Temperature (ºC)	Peak Power Density (W cm^{-2})	References
Atmospheric plasma spraying	BCZYYb(7111)| LSCF	700	0.32	Ref.[1]
WPS	BCZYYb(7111)| BSCF-BCZYYb	600	0.418	Ref.[2]
WPS	$BaZr_{0.16}Ce_{0.64}Y_{0.1}Yb_{0.1}O_{3-\delta}$| BLC	600	0.422	Ref.[3]
WPS	BCZYYb4411| PNC55	600	0.732	Ref.[4]
**WPS**	**BCZYYb| PNC73**	**600**	**0.962**	**This work**

Table S2: Comparison of the obtained peak power density based on the various fabrication process of ~ 10 to 20 μm electrolyte layer.

Fabrication Process	Electrolyte| air electrode	Temperature (ºC)	Peak Power Density (W cm^{-2})	References
Spin coating	BCZYYb(7111)| BZCY-LSCF	600	0.5	Ref.[5]
Spin coating	BCZYYb(6211)| PBSCF	600	0.650	Ref.[6]
WPS	$Sm_{0.2}Ce_{0.8}O_{1.9}$| BSCF	600	0.765	Ref.[7]
Tape casting	BCZYYb4411| BCFZY	600	0.850	Ref.[8]
Tape casting	BCZYYb4411| PNC73	600	0.906	Ref.[9]
**WPS**	**BCZYYb| PNC73**	**600**	**0.962**	**This work**

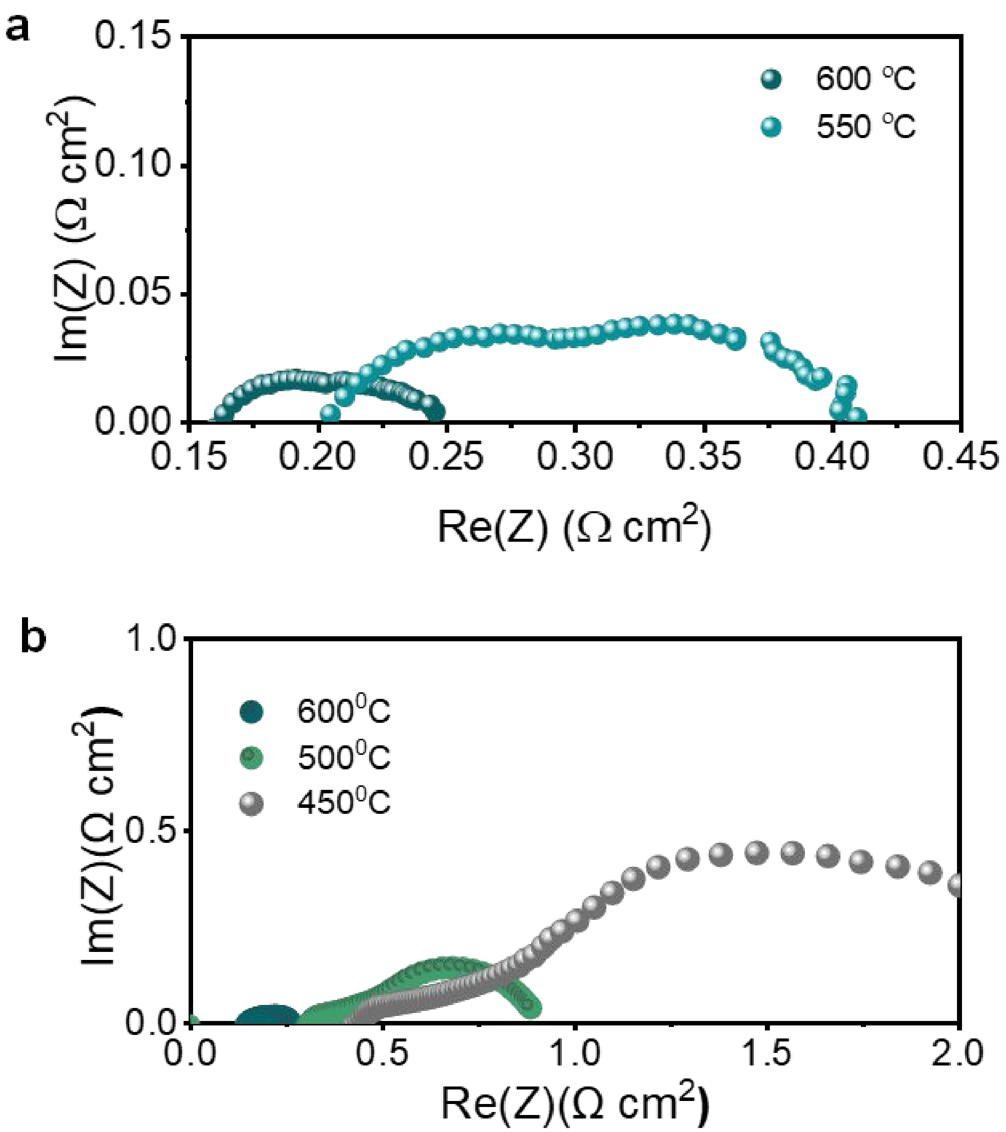

Figure S4. EIS characterization: a) BCZYYb30C at 600 °C and 550 °C. **b)** Comparative EIS analysis of BCZYYb20C on varying the temperature from 450 °C to 600 °C in FC mode, demonstrating that the R_o and R_p are inversely proportional to the applied temperature.

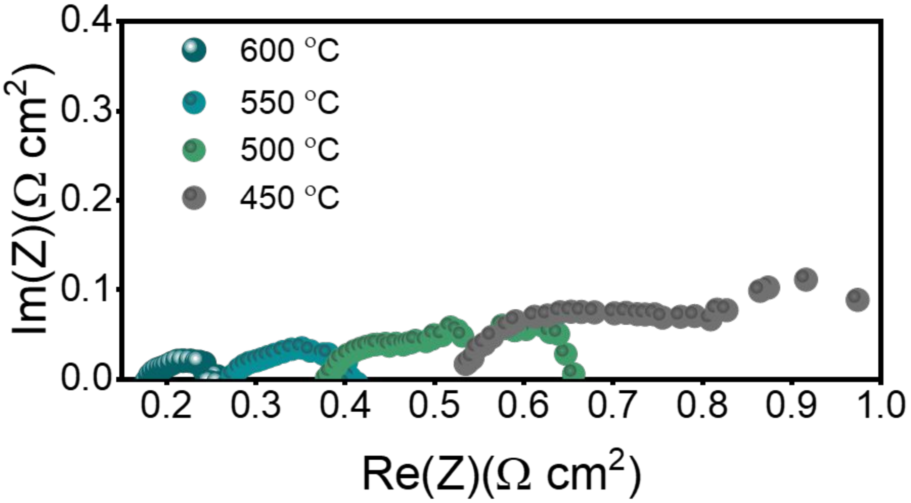

Figure S5. Comparative EIS analysis of BCZYYb20C in EC mode on applying 1.3 V at different temperatures ranging from 450 °C to 600 °C.

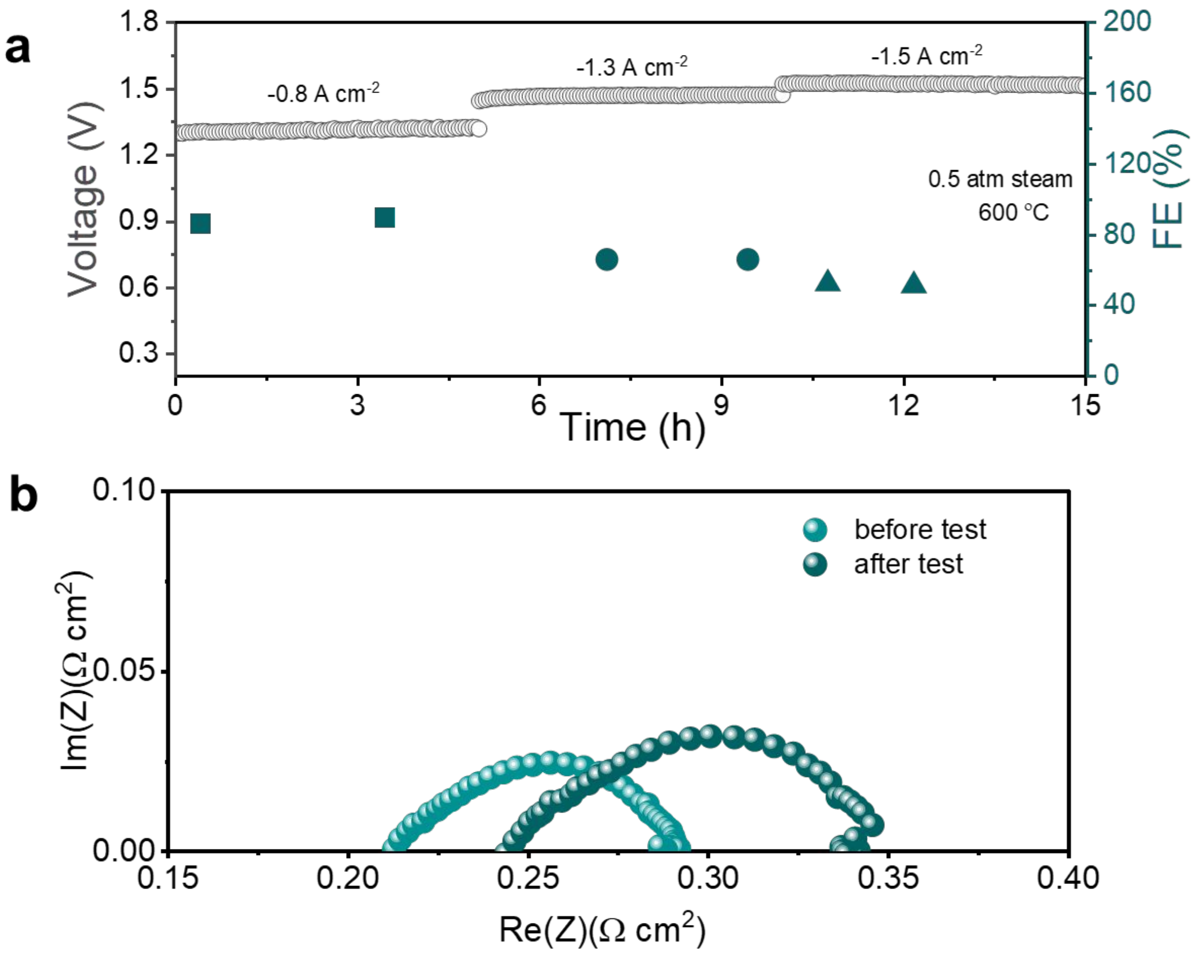

Figure S6. Electrochemical conversion analysis of BCZYYb20C: **a)** Generated voltage was recorded on applying current density of -0.8 A cm^{-2}, -1.3 A cm^{-2}, and -1.5 A cm^{-2} by keeping it constant for 5 h at 600 °C and the corresponding Faradaic efficiency (FE) % was noted respectively. **b)** EIS response of the cell before and after the test.

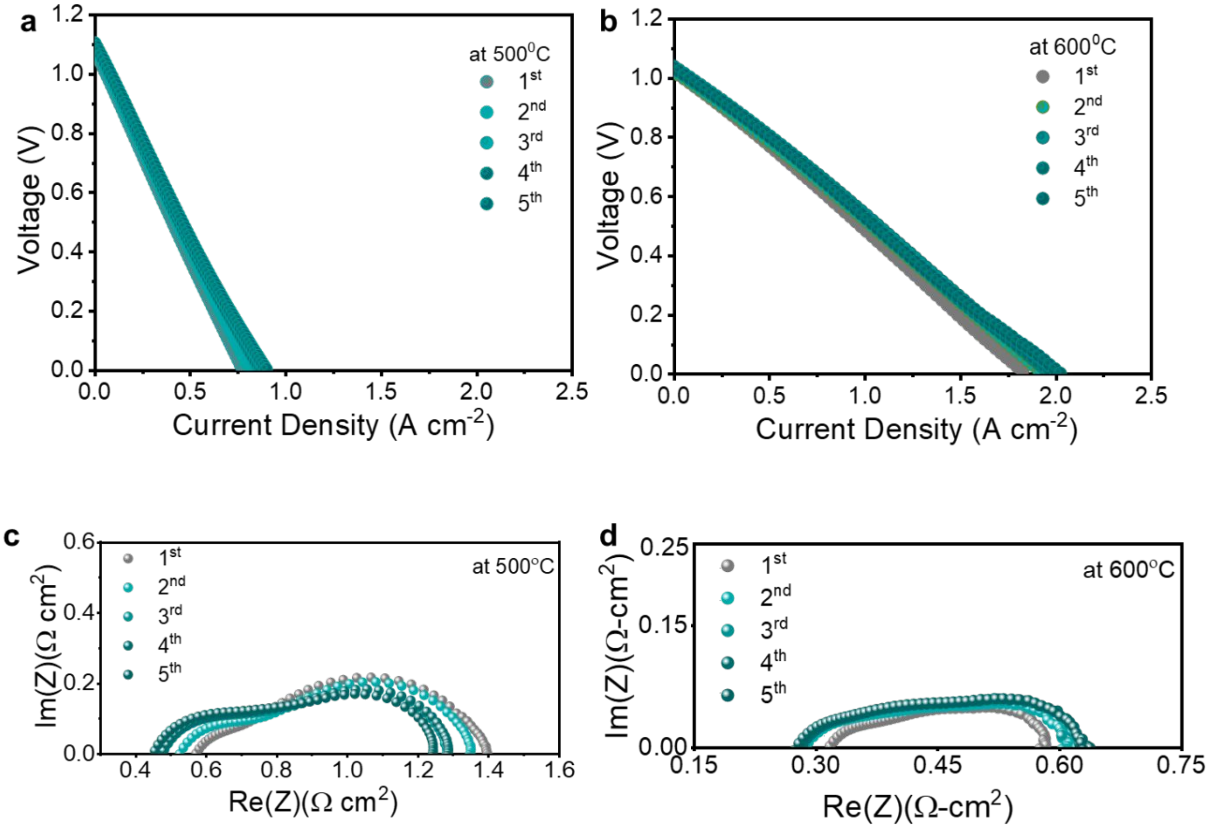

Figure S7. Durability assessment of BCZYYb20C by thermal cyclic performance: a), b) I-V curve at 500 °C and 600 °C, respectively. **c), d)** EIS analysis at 500 °C and 600 °C, respectively, demonstrated an increase in the performance of BCZYYb20C after five cycles due to the decrement in the ohmic resistance.

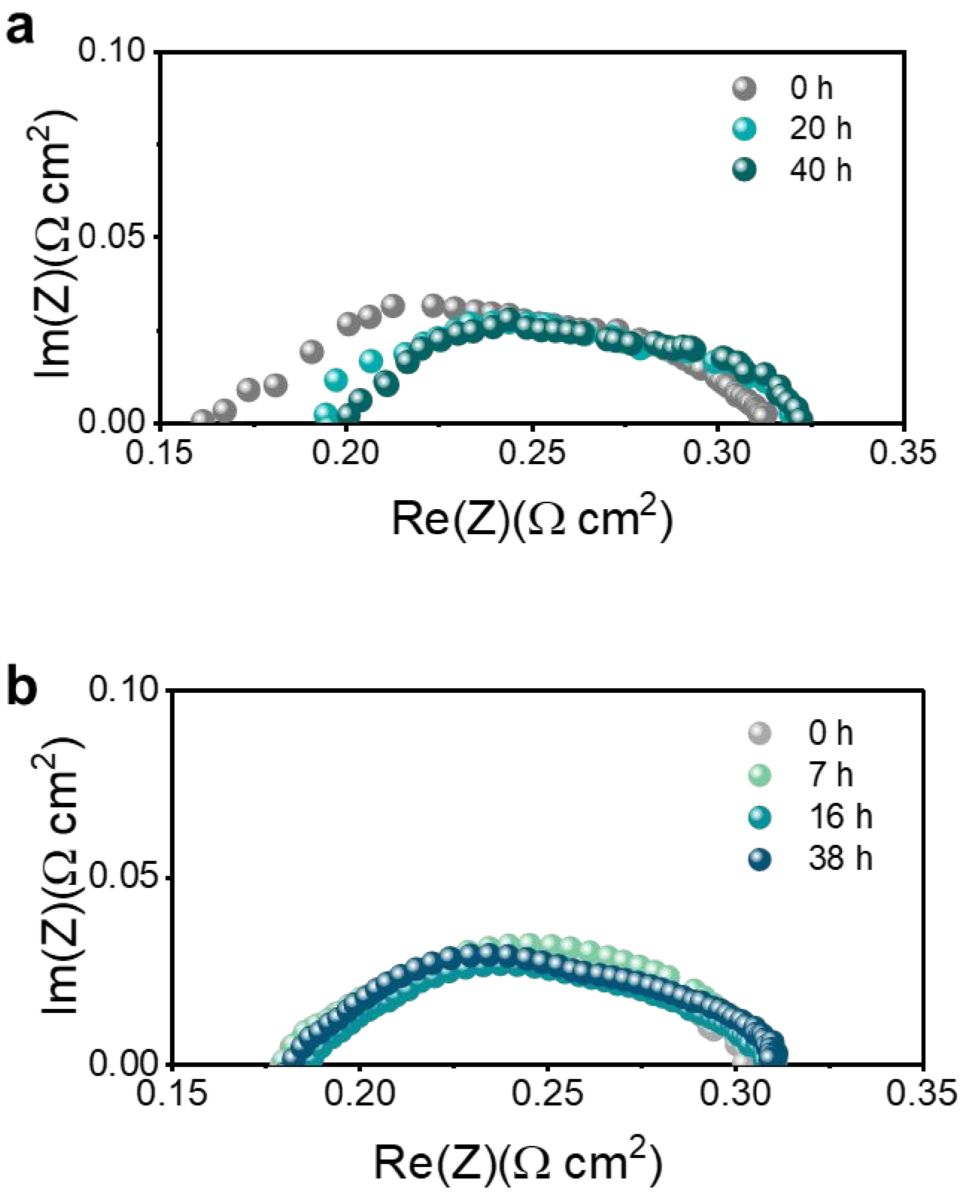

Figure S8. EIS characterization of BCZYYb20C in FC mode: a) During stepwise voltage analysis. **b)** Before and after the transient test.

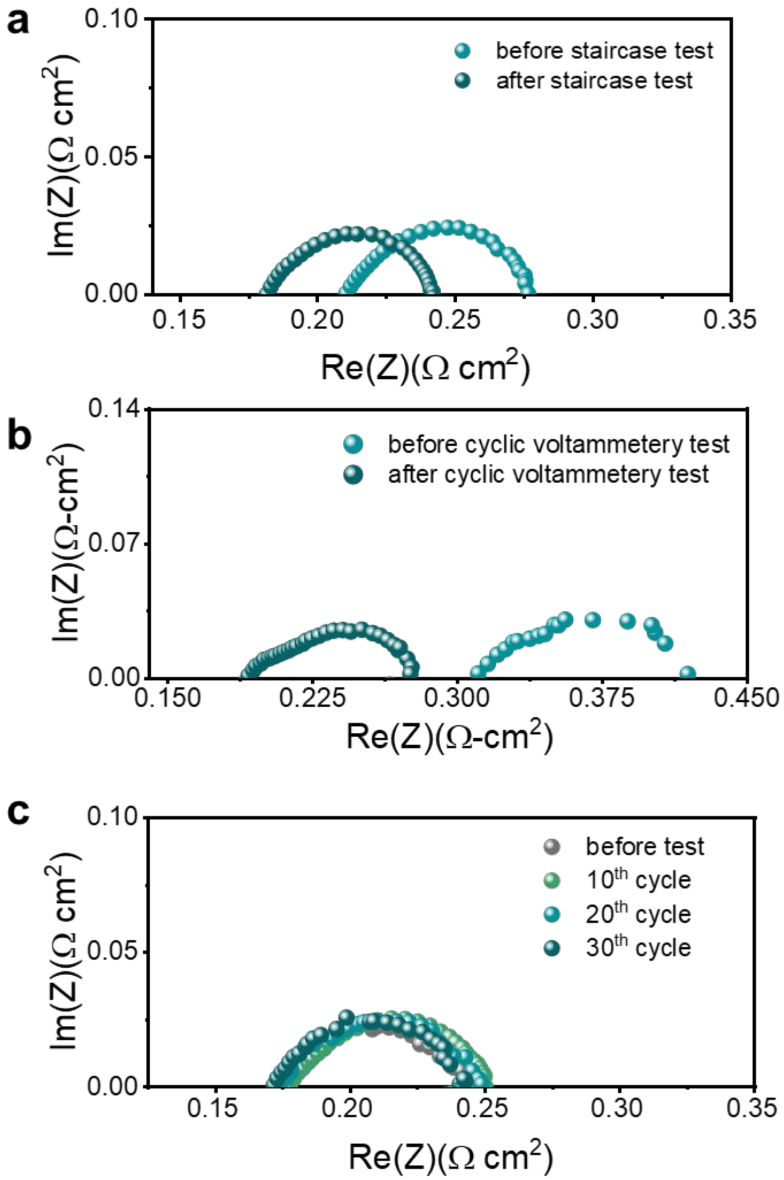

Figure S9. EIS characterization of BCZYYb20C in EC mode: a) Before and after the staircase tests. **b)** Before and after the cyclic-voltammetry test. **c)** During the transient test at 600 °C. In EC mode, EIS characterization was performed by applying 1.3 V to the cell.

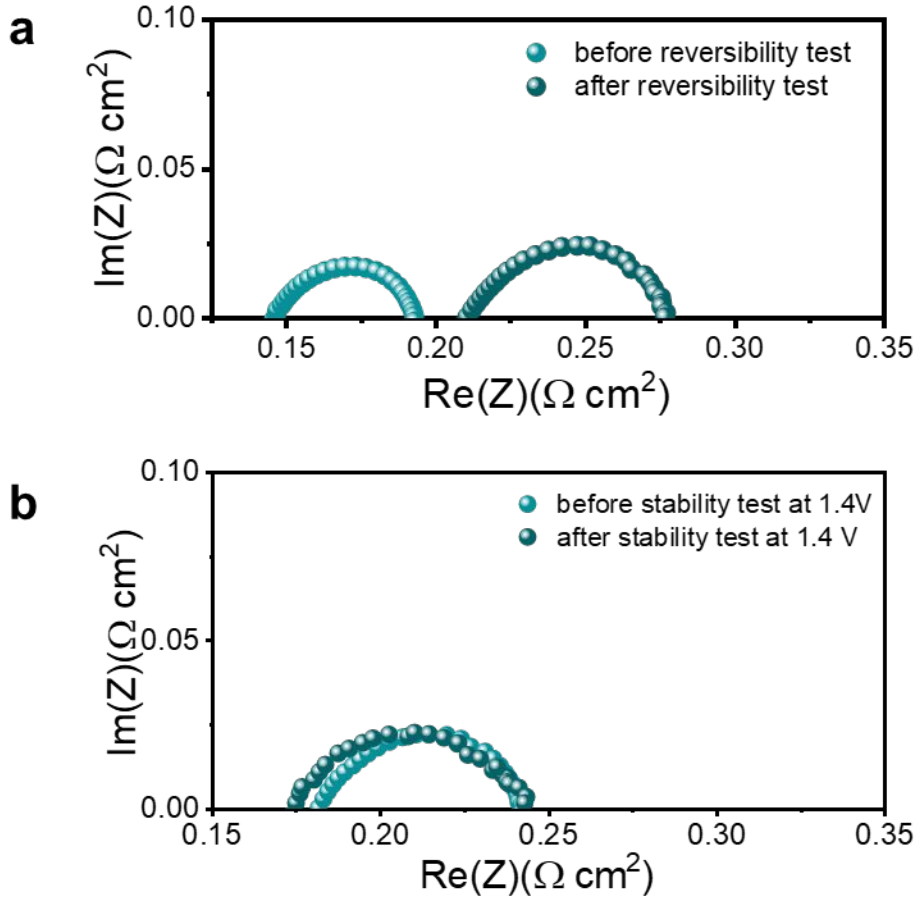

Figure S10: EIS characterization of BCZYYb20C: a) Before and after reversibility test. **b)** Before and after the stability assessment in EC mode, run for 100 h by applying the voltage of 1.4 V.

Table S3: Use of fabricated half-cell for different characterization and electrochemical analysis.

Figure	Characterization	Fabricated Half-cell	Cells tested per test
Figure 1	SEM characterization		
1c		BCZYYb20A	5
1d		BCZYYb20B	5
1e		BCZYYb20C	5
Figure 2	SEM characterization		
2a		BCZYYb10C	3
		BCZYYb20C	3
		BCZYYb30C	3
	AFM characterization		
2b		BCZYYb10C	2
2c		BCZYYb20C	2
2d		BCZYYb30C	2
Figure 3	Electrochemical Test		
3a	I-V-P curve	BCZYYb20C	3
3b	I-V-P curve	BCZYYb30C	3
3g	I-V curve	BCZYYb20C	3
Figure 4	FC mode	BCZYYb20C	
4a	Thermal Cyclic		2
4b	Stepwise test		2
4c	Transient test		2
Figure 5	EC mode analysis	BCZYYb20C	2
Figure 7	SEM characterization	2.5 cm BCZYYb20C	3

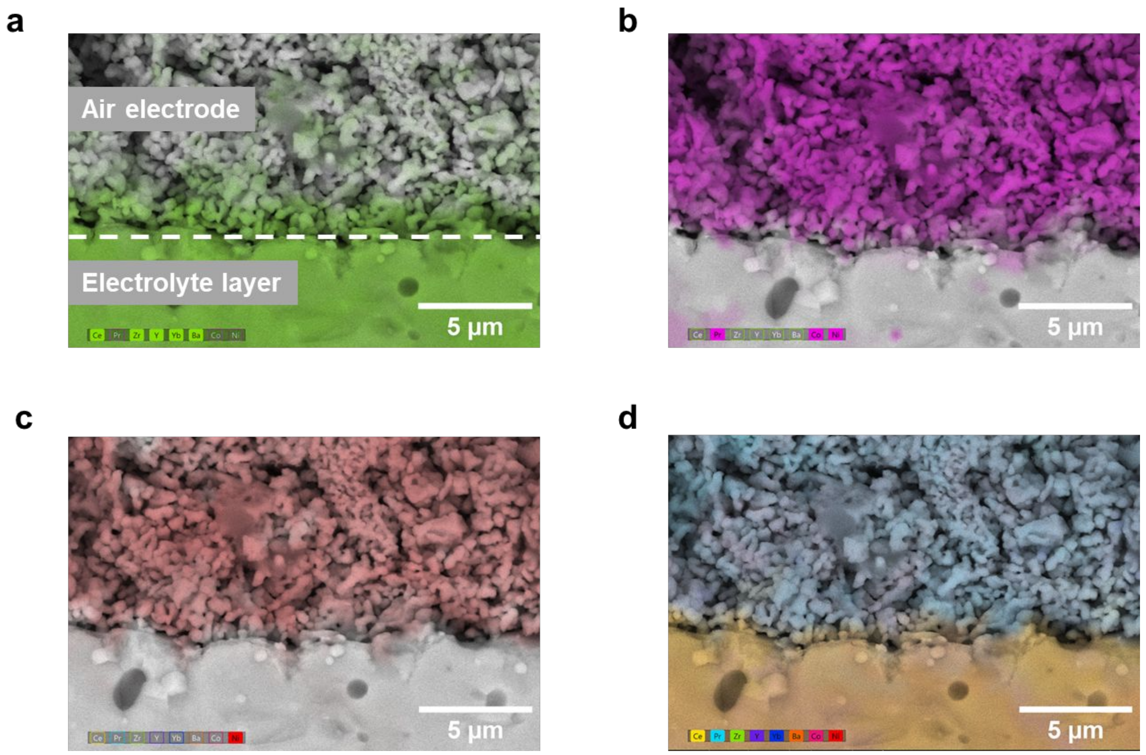

Figure S11. Post-test color-SEM characterization of the cross-section of BCZYYb20C: a) Distribution of Ba, Ce, Zr, Y, and Yb at the interface of the air-electrode and electrolyte. **b)** Pr, Ni, and Co distribution at the interface after electrochemical analysis. **c)** Ni distribution in the cathode layer. **d)** The interface between the air electrode and the electrolyte demonstrated the presence of all elements in their respective layers and the absence of diffusion from one layer to another layer, could be depicted as the tightness and electrochemical stability of the fabricated electrolyte by following the WPS process.

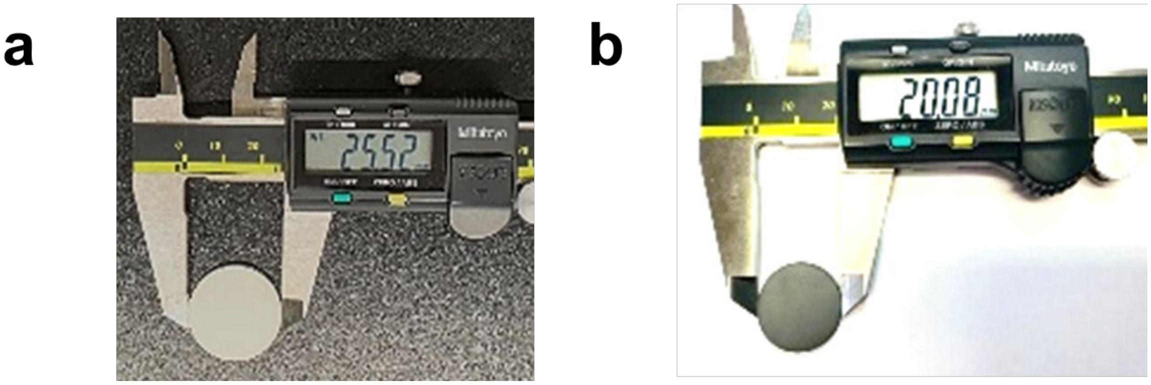

Figure S12. Fabrication of dense electrolyte film on a 2.5 cm diameter anode substrate: a) Pre-sintered BCZYYb20C half-cell. **b)** The fabricated half-cell demonstrated 21% shrinkage after sintering at 1425 °C.

References

(1) Sun, H.; Zhang, S.; Li, C.; Rainwater, B.; Liu, Y.; Zhang, L.; Zhang, Y.; Li, C.; Liu, M. Atmospheric Plasma-Sprayed $BaZr_{0.1}Ce_{0.7}Y_{0.1}Yb_{0.1}O_{3-\delta}$ (BZCYYb) Electrolyte Membranes for Intermediate-Temperature Solid Oxide Fuel Cells. *Ceramics International* **2016**, *42* (16), 19231–19236. https://doi.org/10.1016/j.ceramint.2016.09.088.

(2) Taillades, G.; Pers, P.; Mao, V.; Taillades, M. High Performance Anode-Supported Proton Ceramic Fuel Cell Elaborated by Wet Powder Spraying. *International Journal of Hydrogen Energy* **2016**, *41* (28), 12330–12336. https://doi.org/10.1016/j.ijhydene.2016.05.094.

(3) Zeng, Y.; Kindelmann, M.; Kunafiev, R.; Ivanova, M. E.; Guillon, O.; Leonard, K.; Menzler, N. H. Advancing Proton-Conducting Ceramic Cells with Thin Electrolyte Layers Prepared by Wet Powder Spraying. *Materials Science and Engineering: B* **2025**, *319*, 118340. https://doi.org/10.1016/j.mseb.2025.118340.

(4) Feng, W.; Wu, W.; Jin, C.; Zhou, M.; Bian, W.; Tang, W.; Gomez, J. Y.; Boardman, R.; Ding, D. Exploring the Structural Uniformity and Integrity of Protonic Ceramic Thin Film Electrolyte Using Wet Powder Spraying. *Journal of Power Sources Advances* **2021**, *11*, 100067. https://doi.org/10.1016/j.powera.2021.100067.

(5) Nguyen, N. T. Q.; Yoon, H. H. Preparation and Evaluation of $BaZr_{0.1}Ce_{0.7}Y_{0.1}Yb_{0.1}O_{3-\delta}$ (BZCYYb) Electrolyte and BZCYYb-Based Solid Oxide Fuel Cells. *Journal of Power Sources* **2013**, *231*, 213–218. https://doi.org/10.1016/j.jpowsour.2013.01.011.

(6) Kang, E. H.; Choi, H. R.; Park, J. S.; Kim, K. H.; Kim, D. H.; Bae, K.; B. Printz, F.; Shim, J. H. Protonic Ceramic Fuel Cells with Slurry-Spin Coated $BaZr_{0.2}Ce_{0.6}Y_{0.1}Yb_{0.1}O_{3-\delta}$ Thin-Film Electrolytes. *Journal of Power Sources* **2020**, *465*, 228254. https://doi.org/10.1016/j.jpowsour.2020.228254.

(7) Shi, H.; Zhou, W.; Ran, R.; Shao, Z. Comparative Study of Doped Ceria Thin-Film Electrolytes Prepared by Wet Powder Spraying with Powder Synthesized via Two Techniques. *Journal of Power Sources* **2010**, *195* (2), 393–401. https://doi.org/10.1016/j.jpowsour.2009.07.056.

(8) Kim, D.; Bae, K. T.; Kim, K. J.; Im, H.-N.; Jang, S.; Oh, S.; Lee, S. W.; Shin, T. H.; Lee, K. T. High-Performance Protonic Ceramic Electrochemical Cells. *ACS Energy Lett.* **2022**, *7* (7), 2393–2400. https://doi.org/10.1021/acsenergylett.2c01370.

(9) Zheng, S.; Wu, W.; Zhang, Y.; Zhao, Z.; Duan, C.; Karki, S.; Ding, H. Enhancing Surface Activity and Durability in Triple Conducting Electrode for Protonic Ceramic Electrochemical Cells. *Nat Commun* **2025**, *16* (1), 4146. https://doi.org/10.1038/s41467-025-59477-9.
